# Jinhong Tablet Reduces Damage of Intestinal Mucosal Barrier in Rats with Acute Biliary Infection via Bcl-2/Bax mRNA and Protein Regulation

**DOI:** 10.1155/2017/4985926

**Published:** 2017-10-03

**Authors:** Xiaoqiang Liang, Xiao Ni, YongQi Wang, Jinkun Xie, Xuelin Zhang, Honggang Gu, Jingzhe Zhang

**Affiliations:** Longhua Hospital Affiliated to Shanghai University of Traditional Chinese Medicine, Shanghai 200032, China

## Abstract

**Objective:**

To explore the effects and mechanism of Jinhong Tablet on intestinal mucosal barrier function and SIRS in rats with acute biliary infection.

**Methods:**

36 SD male rats were divided into three groups: sham operation (control), acute biliary infection (ABI) model, and Jinhong Tablet (Jinhong) group. Jinhong group were force-fed with Jinhong Tablet, while the other two groups received oral saline. At days 3 and 5, morphological changes of intestinal mucosa were assessed. Serum diamine oxidase (DAO), D-lactate, and endotoxin levels were measured. And the genes bcl-2 and bax in intestinal tissues were tested by real-time PCR and Western blotting.

**Results:**

Intestinal damage was significantly less severe in Jinhong group compared with ABI group, as indicated by Chiu's scoring, TUNEL analysis, and serum DAO, D-lactic acid, and endotoxin levels. Additionally, the expression of bax mRNA and protein was decreased and the ratio of bcl-2/bax mRNA and protein was increased compared with ABI group.

**Conclusion:**

Jinhong Tablet had a positive intervention on acute biliary infection through improving inflammation and intestinal mucosal barrier, inhibiting excessive apoptosis of intestinal epithelial cells via bax and bcl-2 gene, and protein regulation.

## 1. Introduction

Acute biliary infections (cholecystitis, cholangitis, and biliary pancreatitis) are common diseases in surgery. Severe infections often lead to Systemic Inflammatory Response Syndrome (SIRS), which in turn causes multiple organ failure (MOF) with a high mortality rate [1, 2]. In clinic, there are a variety of treatment methods, including anti-infection, antishock, surgery, and other comprehensive treatment schemes in view of local biliary diseases, but the effect is still unsatisfactory. Severe infection involved in other organs always leads to more serious body damage and the incidence of poor prognosis. In recent years, the importance of intestinal mucosal barrier function in acute biliary infection has been recognized [3, 4]. The gut is considered the driving force behind SIRS [5–7]. Intestinal mucosal barrier can prevent intestinal bacteria and toxins from intestinal mucosa from entering into other organs and blood circulation in the body.

Our previous study demonstrated that there was some intestinal mucosal damage in intestinal epithelial cells in rats with acute biliary infection, such as congestion, edema, and infiltration [8]. Excessive apoptosis of intestinal epithelial cells is the cytological basis in acute biliary infection [9]. There are two main types of apoptosis genes: antiapoptosis gene (e.g., bcl-2) and proapoptotic gene (e.g., bax). When acute biliary infection occurs, the body produces large amounts of cytokines (IL-2, IL-6, IL-8, TNF-*α*, etc.) which can induce excessive apoptosis and impair intestinal mucosal barrier function. Along with ischemia and hypoxia, intestinal mucosal permeability increased. Then the intestinal bacteria and endotoxins shift into the blood and abdominal cavity which can trigger it a second time to stimulate the inflammatory cascade. All these can bring amplification effect to induce SIRS. 

Jinhong Tablet is a traditional Chinese medicine which has treated acute biliary infections for more than 40 years in our hospital. It is composed of* Rheum palmatum *L. stem,* Sargentodoxa cuneata *stem, and* Taraxacum mongolicum*, which has the antipyretic and purgative effects. Our early clinical studies showed that Jinhong Tablet can improve fever, abdominal pain signs, reduce TNF-*α*, IL-6, IL-8, and other inflammatory cytokines, inhibit excessive inflammatory response, and maintain organism's balance between inflammation and anti-inflammatory in severe acute biliary infections. Hence, it can help the affected to recover from the acute phase smoothly [10]. At the same time, our animal experiment has proved that Jinhong Tablet can repair intestinal mucosal damage to some extent [8].

Therefore, we hypothesized that Jinhong Tablet would reduce damage of intestinal mucosal barrier via inhibiting the excessive apoptosis of intestinal epithelial cells through bcl-2/bax mRNA and protein regulation and then block the occurrence of acute biliary infection evolved into SIRS. This study was carried out to verify this hypothesis.

## 2. Materials and Methods

### 2.1. Experimental Drugs

*Escherichia coli* was provided by the microbiology laboratory of Longhua Hospital; Jinhong Tablet (composed of* Rheum palmatum *L. stem,* Sargentodoxa cuneata *stem, and* Taraxacum mongolicum*, 0.3 g/piece) was provided by the Chinese Medicine Pharmacy of the Affiliated Long Hua Hospital of Shanghai University of Traditional Chinese Medicine (TCM).

### 2.2. Animals and Treatments

Fourteen-week-old specific pathogen-freemale Sprague-Dawley rats weighing 200 ± 10 g were purchased from Shanghai Silaike Experimental Animal Co. Ltd. (certification number SCXK (Hu) 2012-0002). Animal experiment ethical approval was obtained from the Affiliated Long Hua Hospital of Shanghai University of Traditional Chinese Medicine. A total of 36 male SD rats were randomly divided into three groups with 12 rats per group (*n* = 12), sham operation group (control), acute biliary infection model group (ABI), and Jinhong Tablet group (Jinhong). Acute biliary infection was induced in rats by pancreatic ductal injection of* E. coli* (5 × 10^9^ CFU/ml) in the latter two groups. In control group, the abdomen was open for 10 minutes and closed without bacterial injection. Jinhong group animals were force-fed with Jinhong Tablet as 0.33 g/kg·d; the dose of rat administration is based on the human mouse equivalent dosage conversion according to the textbook* Pharmacology and Pharmacology Experiment of TCM* (edited by Chen Qi, People's Medical Publishing House Co., Ltd., 1994.), while the other two groups received oral saline. Six rats per group were sacrificed at days 3 and 5, respectively.

### 2.3. Specimen Preparation

Samples were collected after intervention, respectively, at days 3 and 5. Rats were anesthetized by intraperitoneal injection of 4 mg sodium pentobarbital. Then, 3 ml blood was collected from the abdominal aortic; the serum after centrifugation was stored at −80°C. Intestinal tissue specimens (terminal ileum 3 cm) were cut and fixed in 4% paraformaldehyde for 24 h, paraffin-embedded, and sliced at 5 *μ*m sections for H&E staining and TUNEL analysis. Total RNA and protein were isolated from fresh intestinal tissue and stored at −80°C for the follow-up experiment.

### 2.4. Scoring Intestinal Mucosal Injury

Small intestine tissue sections from all three groups were stained with HE staining. The severity of damage was evaluated by two independent, blinded pathologists using Chiu's scoring method [11]. Intestinal mucosal damage was scored from 0 to 5: 0, normal mucosal villi; 1, development of subepithelial Gruenhagen's space at the villus apex; 2, extension of subepithelial space with moderate lifting of the epithelial layer from the lamina propria; 3, massive epithelial lifting on villus sides, possibly with a few denuded tips; 4, denuded villi with the lamina propria and dilated capillaries exposed, possibly with increased cellularity of the lamina propria; 5, digestion and disintegration of the lamina propria, hemorrhage, and ulceration.

### 2.5. TUNEL Analysis of Intestinal Mucosal

Terminal deoxynucleotidyl transferase dUTP nick end labeling (TUNEL) analysis was applied to determine intestinal mucosal cell apoptosis. Briefly, paraffin sections were deparaffinized and rehydrated. Apoptotic cells were detected using the in situ cell death detection kit. Stained slides were washed and sealed with an aqueous mounting medium. For each animal, the number of TUNEL immunoreactive cells was obtained by counting 20 randomly selected fields at 40x magnification from 2 separate sections.

### 2.6. Serum DAO, D-Lactate, and Endotoxin Detection

Serum samples at days 3 and 5 were obtained for DAO, D-lactate, and endotoxin detection, using enzyme-linked immunosorbent assay (ELISA) kits according to the manufacturer's instructions (DAO detection kit was purchased from Nanjing Science and Technology Company and D-lactic acid and endotoxin detection kits were provided by Xiamen Chinese Horseshoe Crab Reagent Factory).

### 2.7. Real-Time Quantitative PCR

Total mRNA was extracted by the Trizol method. RNA of sufficient purity (A260/A280 value 1.8–2) was used for subsequent experimentation. The concentrations were adjusted to 1 *µ*g/*µ*L, and then mRNA was heat-denatured for 5 min at 65°C, cooled immediately, and reverse-transcribed to cDNA using the Sensiscript RT Kit (TAKARA, Japan). Reverse transcription was performed in a 20 *µ*L volume (9 uL nuclease-free water, 4 *µ*L 5x RT Buffer, 1 *µ*L RT Enzyme, 1 *µ*L Oligo dT primer Mix, 1 *µ*L Random 6-mers, and 4 *µ*L RNA). Reaction conditions were as follows: 37°C for 15 min and 85°C for 5 s. Primers were obtained from Shanghai Haojia Gene Company (Shanghai, China) and their sequences were seen in Table 1.

Real-time quantitative PCR and melt-curve analyses were performed with the SYBR Green Real-Time PCR Kit (TAKARA, Japan) and an iCycler machine (Corbett, Rotor Gene 3000, Australia). Amplification was comprised of 40 cycles (95°C for 15 s, 62°C for 20 s, and 72°C for 15 s for bax and bcl-2 and 95°C for 15 s, 56°C for 20 s, and 72°C for 15 s for GAPDH). The relative quantity of the target genes was determined by the comparative Ct value/2^−ΔΔCt^ method with GAPDH as control.

### 2.8. Western Blot Assay

Total protein extracts were isolated after homogenizing. Briefly, equal amounts of proteins (50 *μ*g) were separated by SDS-PAGE and transferred to a nitrocellulose membrane. Membranes were blocked with 2% bovine serum albumin (BSA) and then incubated with appropriate primary antibodies overnight at 4°C. Then an antiworking fluid was added (bax, bcl-2, and GAPDH, provided by Abcam Company, USA) and gently shaken for 4 h. After washing, the membrane was incubated with HRP-labeled goat anti-rabbit Ig G working solution at room temperature. The protein expression was detected by staining with TANON-2008 Gel imaging system. The expression level of protein is expressed by optical density value of target protein/GAPDH.

### 2.9. Statistical Analysis

Statistical analysis was performed using SPSS 18.0 software. Data measurement was expressed as mean values ± standard deviation (SD). Normal distribution and homogeneity of variance assessments were carried out first. For experimental data that met the criteria, comparisons between multiple groups were performed using single-factor analysis of variance (analysis of variance, ANOVA); and comparisons among groups were performed using LSD-*t* (least significant difference-*t*). For experimental data that did not meet the normal distribution and homogeneity of variance criteria, nonparametric tests were adopted. *P* < 0.05 was considered significant.

## 3. Results

### 3.1. Intestinal Morphology

**Figure 1:**
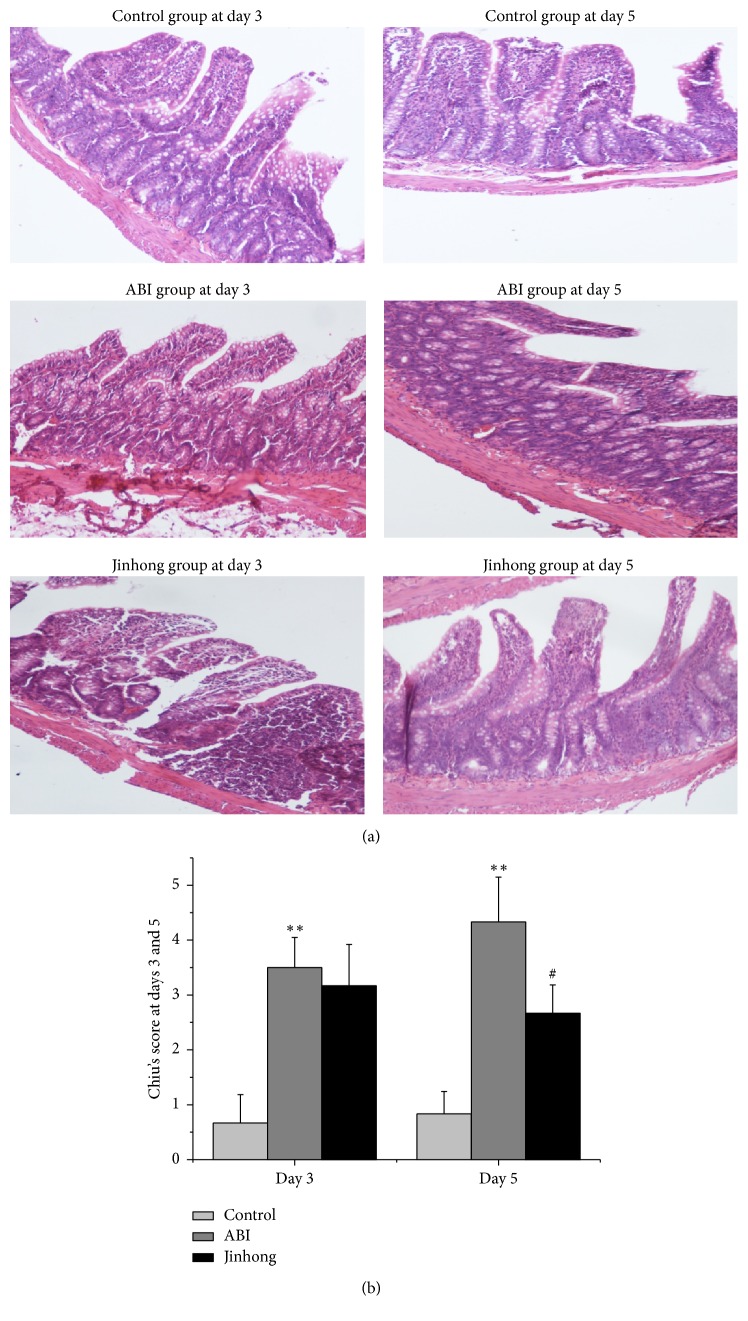
H&E staining: hematoxylin and eosin staining on the intestinal tissue at days 3 and 5, respectively ((a), magnification, ×100), and the quantitative data (b) of Chin's scores. Chin's scores were significantly increased in ABI group compared with control group at days 3 and 5 (*P* < 0.01). And Chin's scores were significantly reduced in Jinhong group compared with ABI group at day 5 (*P* < 0.05). Values were expressed as mean ± SD; *n* = 6 in each group; ^*∗∗*^*P* < 0.01 versus control group; ^#^*P* < 0.05 versus ABI group.

To determine whether the intestinal mucosa was damaged and the improvement of Jinhong Tablet, we performed the morphological changes of the small intestine by HE staining. The results show that there was a more severe damage with Chiu's scores in acute biliary infection group compared with control group at days 3 and 5, respectively (*P* < 0.01) (Figures 1(a) and 1(b)). Compared with acute biliary infection group, the scores were significantly reduced in Jinhong group at day 5 (*P* < 0.05) (Figures 1(a) and 1(b)).

### 3.2. TUNEL Analysis for Apoptosis Assessment in the Intestinal Mucosa

**Figure 2:**
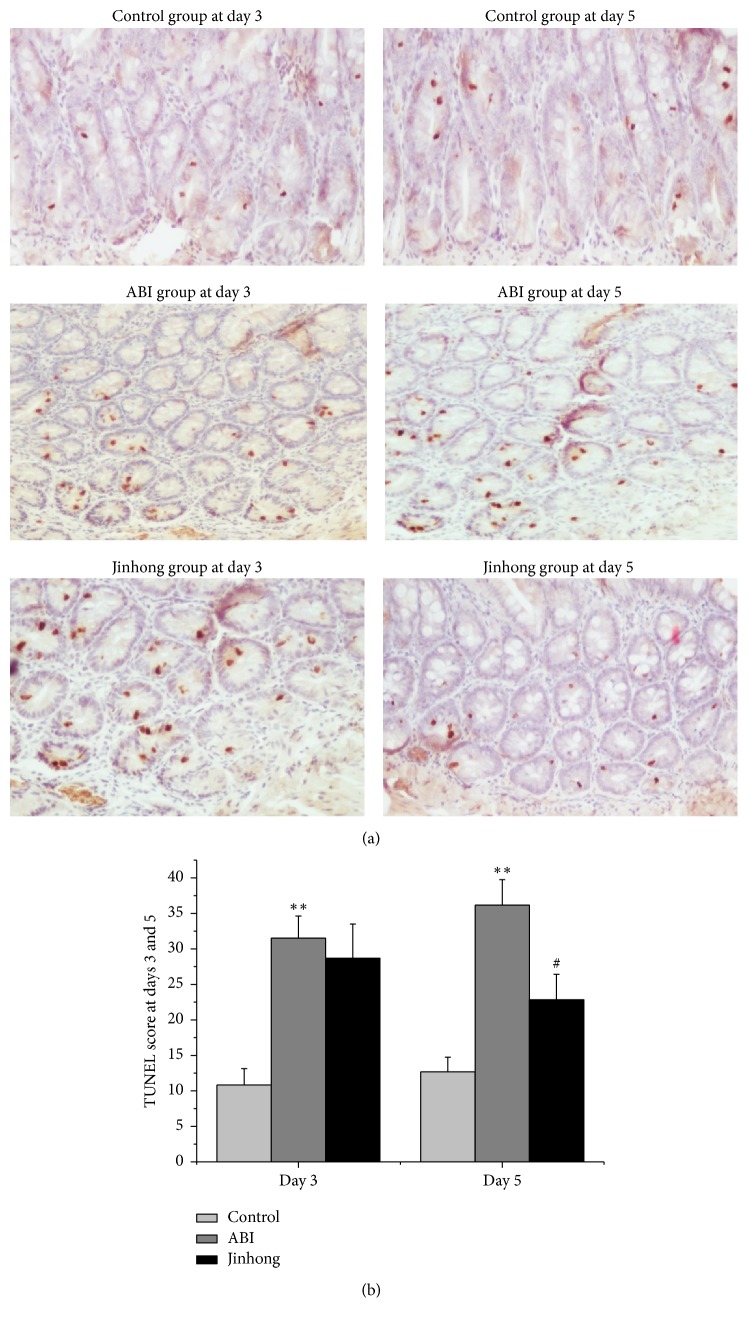
TUNEL analysis: TUNEL staining for apoptosis on the intestinal tissue at days 3 and 5, respectively ((a), magnification, ×100), and the quantitative data (b) of positive cell numbers. TUNEL-positive cell numbers were significantly increased in ABI group compared with control group at days 3 and 5 (*P* < 0.01). And the positive cell numbers were significantly reduced in Jinhong group compared with ABI group at day 5 (*P* < 0.05). Values were expressed as mean ± SD; *n* = 6 in each group; ^*∗∗*^*P* < 0.01 versus control group; ^#^*P* < 0.05 versus ABI group.

To determine whether there was excessive apoptosis of intestinal mucosa epithelial cells, we performed apoptosis of intestinal tissue by TUNEL. The results show that there were more TUNEL-positive cells detected in acute biliary infection group compared with control group at both days 3 and 5 (*P* < 0.01). And significantly fewer TUNEL-positive cells were detected in Jinhong group compared with acute biliary infection group only at day 5 (*P* < 0.01) (Figure 2).

### 3.3. Serum DAO, D-Lactic Acid, and Endotoxin Levels

To confirm the impairment of the intestinal mucosal barrier in acute biliary infection group again and verify the curative effect of Jinhong Tablet, we examined the serum levels of DAO, D-lactic acid, and endotoxin by enzyme-linked immunosorbent assay (ELISA) kits according to the manufacturer's instructions. The results show that serum DAO, D-lactic acid, and endotoxin levels in acute biliary infection group were significantly increased compared with control group at both days 3 and 5 (*P* < 0.01). And serum DAO, D-lactic acid, and endotoxin levels in Jinhong group were decreased at both days 3 and 5 compared with acute biliary infection group (*P* < 0.01) (Tables 2 and 3).

### 3.4. Bcl-2 and Bax mRNA Expression in Intestinal Tissues at Days 3 and 5

**Figure 3:**
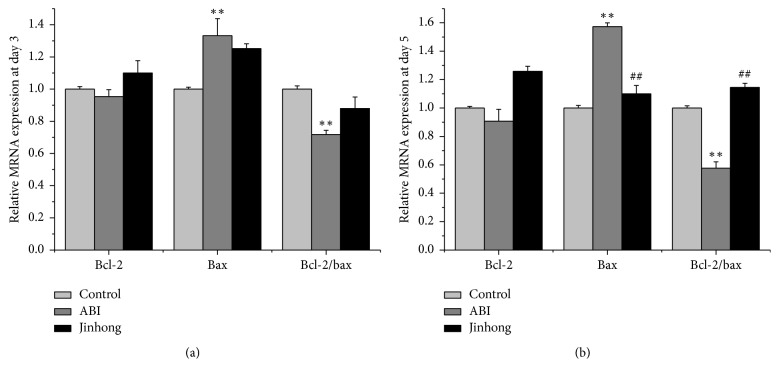
Relative mRNA expression analysis: mRNA expression of bcl-2, bax in intestinal tissue, and bcl-2/bax mRNA expression relative ratio at day 3 (a) and day 5 (b). Values were expressed as mean ± SD; *n* = 6 in each group; ^*∗∗*^*P* < 0.01 versus control group; ^##^*P* < 0.01 versus ABI group.

To further verify the antiapoptotic effect of Jinhong Tablet and to determine whether the effect might be explained by alterations in the expression of the apoptosis genes bcl-2 and bax, we performed real-time PCR. The results show that, at day 3 (Figure 3(a)), the expression of bax mRNA in acute biliary infection group was enhanced compared with control group (*P* < 0.01) and bcl-2/bax was decreased (*P* < 0.01). Compared with acute biliary infection group, there was no significant difference of bax mRNA expression and bcl-2/bax ratio in Jinhong group.

At day 5 (Figure 3(b)), compared with control group, the expression of bax mRNA in acute biliary infection group was enhanced (*P* < 0.01) and bcl-2/bax was decreased (*P* < 0.01). Compared with acute biliary infection group, bax mRNA expression in Jinhong group was attenuated (*P* < 0.01) and bcl-2/bax ratio in Jinhong group was raised (*P* < 0.01)

### 3.5. Bcl-2 and Bax Protein Expression in Intestinal Tissues at Days 3 and 5

**Figure 4:**
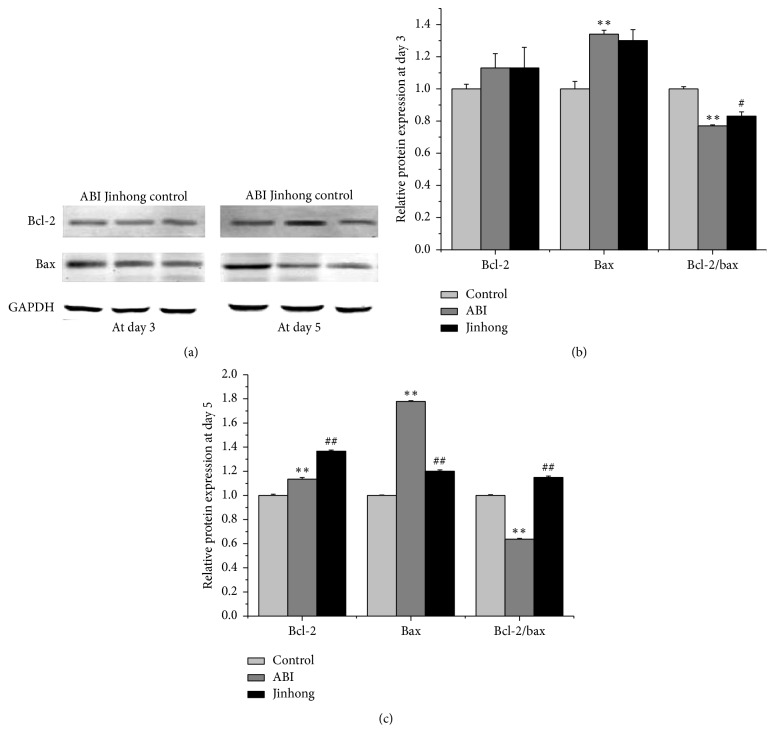
Relative protein expression analysis: protein expression of bcl-2 and bax in intestinal tissue at days 3 and 5, respectively (a), and the densitometric quantification of the protein expression levels, which are expressed as a ratio to the expression of GAPDH of bcl-2, bax, and bcl-2/bax protein expression ratio at day 3 (b) and day 5 (c). Values were expressed as mean ± SD; *n* = 6 in each group; ^*∗∗*^*P* < 0.01 versus control group; ^#^*P* < 0.05, ^##^*P* < 0.01 versus ABI group.

The protein expression of bcl-2 and bax was further assessed by Western blotting. At day 3 (Figures 4(a) and 4(b)), compared with control group, bax protein expression in acute biliary infection group was enhanced (*P* < 0.01) and bcl-2/bax was decreased (*P* < 0.01). Compared with acute biliary infection group, bcl-2/bax ratio in Jinhong group was enhanced (*P* < 0.05).

At day 5 (Figures 4(a) and 4(c)), compared with control group, bcl-2 and bax protein expression in acute biliary infection group was enhanced (*P* < 0.01) and bcl-2/bax was decreased (*P* < 0.01). Compared with acute biliary infection group, bcl-2 protein expression in Jinhong Tablet group was enhanced (*P* < 0.01), bax protein expression in Jinhong Tablet group was reduced (*P* < 0.01), and bcl-2/bax ratio in Jinhong group was raised (*P* < 0.01).

## 4. Discussion

In recent years, the importance of intestinal mucosal barrier function in acute biliary infection has been recognized. Clinical studies revealed that bacterial infections, necrosis, MODS, and MOF induced by acute pancreatitis are closely related to intestinal mucosal barrier dysfunction in early disease stage. It is suggested that intestinal mucosal barrier function is an important factor which affects the prognosis of acute biliary infection [12–14].

DAO is primarily expressed in small intestine and rarely detected under normal circumstances [15, 16]. When intestinal injury occurs, tissue DAO levels decrease, while serum DAO amounts increase [17, 18]. D-lactic acid is a metabolic product of intestinal bacteria. Serum D-lactic acid levels can reflect the intestinal mucosal barrier function easily because their change is related to intestinal mucosal barrier damage closely [19, 20]. LPS is the main pathogenic component of endotoxins which released cytokines and other inflammatory mediators in the disease. Intestinal absorption of endotoxins increases with intestinal dysfunction, resulting in increased blood endotoxin levels, which in turn aggravated intestinal mucosal barrier damage [21, 22].

Our results show that there was some damage on intestinal mucosa in acute biliary infection group, and Jinhong Tablet can improve the damage to some degree. Meanwhile, Chiu's scoring system results also showed a significantly higher severity of intestinal mucosal damage in acute biliary infection group compared with control group at days 3 and 5, and Jinhong Tablet can significantly decrease the severity compared with acute biliary infection group at day 5.

It is known that TUNEL assay is a quantitative method for apoptosis [23, 24]. So we performed apoptosis of intestinal tissue by TUNEL. And the results showed that intestinal mucosa injury in acute biliary infection group was significantly higher compared with control group at days 3 and 5, demonstrating the importance of intestinal mucosa injury in the progress of SIRS occurrence. Meanwhile, there were fewer TUNEL-positive cells in Jinhong group compared with acute biliary infection group at day 5 (*P* < 0.01); what is more, serum indexes DAO, D-lactic acid, and endotoxin also showed a higher level in acute biliary infection group and a lower level in Jinhong group (*P* < 0.01), also clearly suggesting that Jinhong Tablet overtly reduced intestinal mucosal barrier damage.

As we know, bax and bcl-2 are typical genes closely related to apoptosis; the former promotes apoptosis and the latter inhibits apoptosis [25]. Therefore, the expression of bax and bcl-2 genes and proteins was also measured. Our results show that, at days 3 and 5, bax mRNA and protein expression in acute biliary infection group was enhanced and the ratio of bcl-2/bax was decreased compared with control group, which again demonstrated that there was excessive apoptosis of intestinal epithelial cells in acute biliary infection group. After the intervention of Jinhong Tablet, there was no significant difference of bax mRNA and protein expression and bcl-2/bax ratio compared with acute biliary infection group at day 3. But at day 5, bax mRNA and protein expression decreased and bcl-2/bax ratio was increased compared with acute biliary infection group. This indicated that although Jinhong Tablet had no effect on intestinal epithelial cell apoptosis at day 3, it can block the nonphysiological excessive apoptosis at day 5. This may be related to the slow effects of traditional Chinese medicine.

Overall, the importance of “second infectious attack following the primed condition” has been emphasized as an aggravating factor [26, 27]. Protecting intestinal mucosal barrier and preventing bacteria and endotoxins from shifting into the blood may be one way to avoid SIRS occurrence and improve disease prognosis. Thus, based on the data in the present study, the formula Jinhong Tablet had a positive intervention on acute biliary infection through improving inflammation and intestinal mucosal barrier, inhibiting excessive apoptosis of intestinal epithelial cells via bax and bcl-2 gene, and protein regulation. As for the signaling pathway and upstream target of Jinhong Tablet on inhibiting of intestinal mucosal epithelial cells' apoptosis which is more concentrated on MAPK signal transduction pathway [28, 29], we will make a further experiment to deeply prove that in future research.

## Figures and Tables

**Table 1:** Primer sequence of bax, bcl-2, and GAPDH.

Gene	Sequence (5′-3′)	Product length (bp)
Bax	F: AGACACCTGAGCTGACCTTGGAG	197
	R: GTTGAAGTTGCCATCAGCAAACA	
Bcl-2	F: TGAACCGGCATCTGCACAC	116
	R: CGTCTTCAGAGACAGCCAGGAG	
GAPDH	F: GGAGATTACTGCCCTGGCTCCTA	150
	R: GACTCATCGTACTCCTGCTTGCTG	

**Table 2:** Serum DAO, D-lactic acid, and endotoxin levels at day 3 (mean ± SD).

Group	DAO (U/L)	D-Lactic acid (mmol/l)	Endotoxin (EU/ml)
Control	13.68 ± 0.93	0.30 ± 0.06	0.43 ± 0.01
ABI	80.84 ± 1.79^*∗∗*^	4.67 ± 0.12^*∗∗*^	0.60 ± 0.03^*∗∗*^
Jinhong	60.19 ± 1.92^##^	3.73 ± 0.25^##^	0.51 ± 0.02^##^

DAO, D-lactic acid, and endotoxin values at day 3 were expressed as mean ± SD; *n* = 6 in each group. ^*∗∗*^*P* < 0.01 versus control group; ^##^*P* < 0.01 versus ABI group.

**Table 3:** Serum DAO, D-lactic acid, and endotoxin levels at day 5 (mean ± SD).

Group	DAO (U/L)	D-Lactic acid (mmol/l)	Endotoxin (EU/ml)
Control	12.18 ± 1.36	0.28 ± 0.06	0.42 ± 0.02
ABI	51.05 ± 1.99^*∗∗*^	6.53 ± 0.48^*∗∗*^	0.71 ± 0.04^*∗∗*^
Jinhong	39.87 ± 2.77^##^	2.88 ± 0.24^##^	0.45 ± 0.02^##^

DAO, D-lactic acid, and endotoxin values at day 5 were expressed as mean ± SD; *n* = 6 in each group. ^*∗∗*^*P* < 0.01 versus control group; ^##^*P* < 0.01 versus ABI group.
